# Shade tolerance as a key trait in invasion success of submerged macrophyte *Cabomba caroliniana* over *Myriophyllum spicatum*

**DOI:** 10.1002/ece3.9306

**Published:** 2022-09-16

**Authors:** Gergő Koleszár, Balázs András Lukács, Péter Tamás Nagy, Sándor Szabó

**Affiliations:** ^1^ Wetland Ecology Research Group Centre for Ecological Research, IAE Debrecen Hungary; ^2^ Department of Biology University of Nyiregyhaza Nyiregyhaza Hungary; ^3^ Institute of Water and Environmental Management University of Debrecen Debrecen Hungary

**Keywords:** alien, aquatic plant, competition, light, nutrient, temperature

## Abstract

The synergy between climate change, eutrophication, and biological invasion is threatening for native submerged plants in many ways. The response of submerged plants to these changes is a key factor that determines the outcome of biological invasion. In order to explain the invasion successes, we investigated the combined effects of climate change and eutrophication‐related environmental factors (temperature, light, and nutrients) on the trait responses of a native (*Myriophyllum spicatum*) and an alien (*Cabomba caroliniana*) submerged species. In a factorial design, we cultivated the two species in aquaria containing low (0.5 mg N L^−1^, 0.05 mg P L^−1^) and high (2 mg N L^−1^, 0.2 mg P L^−1^) nutrient concentrations, incubated at four light intensities (average 25, 67, 230, and 295 μmol m^−2^ s^−1^ PAR photon flux density) under two temperature levels (21.5 and 27.5 ± 0.5°C). We used four invasion‐related functional traits (relative growth rate (RGR), specific leaf area (SLA), leaf dry matter content (LDMC), and nitrogen to carbon ratio (N:C molar ratio)) to measure the environmental response of the species. We calculated plasticity indexes to express the trait differences between species. *Cabomba caroliniana* showed significantly higher RGR and SLA than *M. spicatum* especially under low light intensity indicating that *Cabomba* is much more shade tolerant. Elevated temperature resulted in higher SLA and reduced LDMC for *C. caroliniana* indicating that *Cabomba* may have higher invasion success. *Myriophyllum* showed higher LDMC than *C. caroliniana*. Chemical analyses of the plant tissue revealed that although *M. spicatum* showed significantly higher N:C molar ratio, nonetheless, the daily nitrogen uptake of *C. caroliniana* was more than three times faster than that of *M. spicatum*. Results supported the idea that due to its higher shade tolerance and nitrogen uptake capacity, *Cabomba* likely has greater invasion success with increasing temperature combined with low light levels.

## Graphical abstract

We investigated the combined effects of climate change and eutrophication‐related environmental factors (temperature, light, and nutrients) on the hard trait responses of a native and an alien submerged species (*Myriophyllum spicatum* and *Cabomba caroliniana*) from the same growth form. Our laboratory experiment clearly pointed out that invasion success of the alien *C. caroliniana* is higher than that of *M. spicatum*, due to its better shade tolerance.

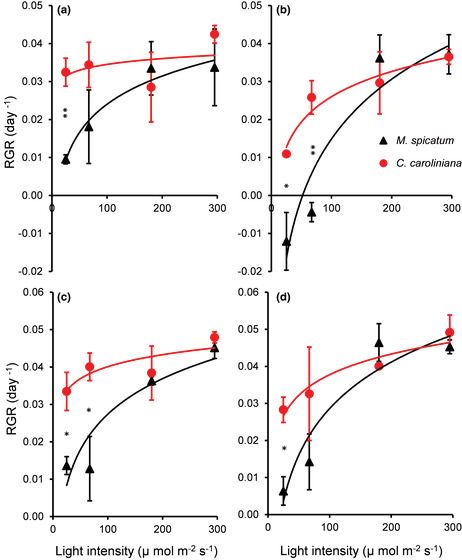

## 1 INTRODUCTION

The introduction of alien plant species and climate change are among some of the major global biodiversity threats. Climate change could alter almost every facet of biological invasion and every interaction between environmental stressors, thereby decreasing the resistance to invasion of natural communities (Dukes & Mooney, 1999; Hellman et al., 2007; Netten et al., 2010). Climate change is also stressing native species to the point of being unable to compete against new invasives (Rejmanek & Richardson, 1996). Moreover, in freshwaters, it intensifies the symptoms of eutrophication (Jeppesen et al., 2010), and a 2–4°C increase in water temperature is forecast by 2100 (Pachauri et al., 2014).

The synergy between climate change and eutrophication is threatening for submerged plants in many ways (Moss, 2011). On one hand, there is a positive correlation between elevated temperature and dominance of free‐floating vegetation over submerged plants (Peeters et al., 2013). In addition, high nutrient concentration of the water may also result in the dominance of phytoplankton (Scheffer & van Nes, 2007) and free‐floating plants (Scheffer et al., 2003; Smith, 2014; Szabó et al., 2022). Both phenomena cause light limitation (Lewis & Bender, 1961; Morris et al., 2003; Phillips et al., 2016), which negatively affects the growth of submerged plants. The response of submerged plants to these changes (i.e., lower light intensity, higher nutrient concentration, and elevated temperature) is a key factor that determines the outcome of biological invasion.

In the naturalization phase of invasion, alien species must adapt to the new environmental conditions in order to establish, survive, and reproduce (Richardson & Pyšek, 2012). Morphological or physiological characteristics (i.e., specific leaf area (SLA), dry matter content, relative growth rate (RGR), and nutrient uptake) that provide a competitive advantage to alien plants over native species have a key role in this process (Vilà & Weiner, 2004). Greater phenotypic differences between alien and native aquatic plants considerably increase the probability of the alien's success (Lake & Leishman, 2004). Accordingly, higher RGR, nutrient uptake, SLA, and higher phenotypic plasticity can contribute to the success of alien aquatic plants (Geng et al., 2006; Lukács et al., 2017; Szabó et al., 2019, 2020). Light, nutrient availability, and temperature are strongly related to climate change and eutrophication. Responding to these environmental factors, invasive plants need to be able to change their phenotypic properties more quickly, such as elongation (Molnár et al., 2015; Szabó et al., 2019), branching, root‐shoot ratio (Szabó et al., 2019), chlorophyll concentration (Szabó et al., 2020), leaf area (Riis et al., 2010), or dry matter content (Larson, 2007). The responses to these factors separately have been well documented; however, there is not a single study addressed to evaluate their combined impact on morphological and physiological traits of submerged macrophytes.

*Cabomba caroliniana* is one of the macrophytes that gains high invasion risk in freshwaters (Matthews et al., 2017). Significant differences were discovered between *C. caroliniana* and native beds for underwater light conditions, macrophyte equitability, and epiphytic algal biomass (Hogsden et al., 2007). Roijackers (2008) stated that growth form plays an important role in the competitive ability of *Cabomba caroliniana*. Specifically, where there are plants with a similar growth form (e.g., *Myriophyllum spicatum* and *M. heterophyllum*), the growth potential of *C. caroliniana* appears to be limited. However, limited information was found on the effects of *C. caroliniana* on native aquatic plants (Matthews et al., 2013).

In our experimental study, we compared the phenotypic plasticity of *Cabomba caroliniana* and *Myriophyllum spicatum*, two submerged aquatic plants with similar growth form (i.e., Myriophyllid: anchored submerged plants with long stems and finely divided submerged leaves, Wiegleb, 1991). *Cabomba caroliniana* (Cabombaceae) is a fast‐growing submerged aquatic plant, native in Argentina, Brazil, Uruguay, Paraguay, and South‐eastern USA (Ørgaard, 1991); however, it forms densely vegetated stands in European freshwaters as an alien species; frequently distributed in the UK (Stace, 1997), the Netherlands (van der Velde et al., 2002), and Hungary (Lukács et al., 2014; Steták, 2004). *Myriophyllum spicatum* (Haloragaceae) is native in Europe, Asia, and North‐Africa (Patten, 1954). We have chosen to compare the environmental response of these species because they frequently occur in the same habitat in Hungary, and we suggest strong competitive interaction between them due to the same growth form display similar extent of realized niche (Begon et al., 1996).

Specific leaf area, leaf dry matter content (LDMC), nitrogen/carbon (N:C) molar ratio, and RGR are considered as the so‐called “response” traits in the plant trait literature (Engelhardt, 2006). It means that it can be used to examine the manner in which biota responds to changes in the environment; therefore, we investigated these traits in order to get answers to our questions.

We hypothesized (H1) that differences in their growth rate and nutrient uptake become more pronounced both under higher temperature or higher nutrient concentration and with decreasing light conditions. We suggest that these factors may contribute to the invasion success of *Cabomba* over *Myriophyllum*. We also hypothesized (H2) that along various environmental conditions, *Cabomba* has a higher phenotypic plasticity than *Myriophyllum* and this may also contribute to its invasion success.

This study aims to evaluate these hypotheses by investigating the combined effects of temperature, light, and nutrients (N, P) on the trait responses of the two species in a laboratory experiment. A further aim is to clarify the limited competition ability of *Cabomba* growing next to *Myriophyllum*, and how this competition is altered by changing environmental conditions. Since submerged species may strongly change light conditions if they are grown in cocultures (Szabó et al., 2019), we cultivated them separately in order to eliminate these effects.

## 2 METHOD

### 2.1 Plant collection, preincubation

We collected apical shoots of *Cabomba caroliniana* from the thermal outflow of Lake Hévíz (N 46.786986°, E 17.194127°), and *Myriophyllum spicatum* from the Eastern Principal Channel (N 47.860911°, E 21.382270°), Hungary. Shoots were preincubated in plastic boxes containing 20 L of deionized water which was supplemented by Na^+^, K^+^, Ca^2+^, Mg^2+^, ${\mathrm{HCO}}_{3}^{-}$, ${\mathrm{SO}}_{4}^{2-}$, and Cl^−^ as general purpose culture solution medium detailed by Barko and Smart (1985). Final concentration of the solutions for nutrients varied from eutrophic (0.5 mg N L^−1^ and 0.05 mg P L^−1^) to hypertrophic (2 mg N L^−1^ and 0.2 mg P L^−1^) through the treatment of adding NH_4_NO_3_ and K_2_HPO_4_ stock solutions (1000 mg L^−1^ for N and for P) to the medium. The nutrient (nitrogen and phosphorus) concentration of medium was set to these values because we intend to simulate the natural conditions and these two trophic levels refers to eutrophic and hypertrophic status of natural waters. The supply of micronutrients was ensured by adding TROPICA Supplier micronutrient solution (Szabó et al., 2010). We preincubated the selected apical shoots (10–12 cm length) for 14 days under 230 μmol m^−2^ s^−1^ photosynthetically active radiation (PAR), 16:8 h L/D regime at 24.5 ± 0.5°C. We renewed the culture medium twice a week. Shoot length nearly doubled (22 cm) under 14 days of preincubation. Therefore, most of the selected apical shoots (11–14 cm) had developed already under preincubation period; hence, they were well accommodated for experimental condition. Before starting the experiment, we removed water from the surface of the plants using a centrifuge (600 RPM, 10 s). We measured subsamples of initial shoots of each species from each nutrient concentration for fresh weight (FW) and dry weight (DW) (*W*_0_).

### 2.2 Laboratory experiment

We placed six apical *Cabomba* and *Myriophyllum* shoots (11–14 cm length, 7.40 ± 0.2 g FW each) separately in 2‐L aquaria (height: 11.5 cm, width: 11.5 cm, length: 18 cm) containing the culture media described above. The initial pH of the water was adjusted to 7.3. Plant shoots were placed free into the aquaria, and they were not planted into a substrate. They were positioned that way to exclude the self‐shading effect. We covered the sides of the aquaria with black foil to avoid light penetration from the sides. For both species, two different nutrient treatments (0.5 mg N L^−1^, 0.05 mg P L^−1^; 2 mg N L^−1^, 0.2 mg P L^−1^) were incubated at four different light intensities varying from strongly shaded to well‐illuminated conditions: 22–28 (L1), 52–82 (L2), 170–290 (L3), and 260–330 (L4) μmol m^−2^ s^−1^ PAR photon flux density. The highest light intensity used in the experiment is roughly the same that we could measure on a summer sunny day under half‐shaded conditions in a natural water. The other light intensities were set to this values because the differences between light treatments were suitable for measuring the effects of light intensity on the plants. The light intensity of lamps was not dimmable, but the distance between the lamps and aquaria was adjustable; therefore, light intensity could be modified in a specific area. We measured the light intensity at the water surface of each aquaria, and they were placed into a lane where the light intensity was appropriate for the treatment. The plants were grown under moderately cold and warm water (21.5 and 27.5 ± 0.5°C) using a controlled temperature water bath. We renewed the culture medium on days 2, 4, and 6. Illumination was carried out by 400 W metal halogen lamps. Each treatment (2 × 2 × 2 × 4 = 32) was replicated three times meaning that we used 96 aquaria. We finished the experiment after 8 days in order to keep the initial light levels more or less constant, as well as to avoid overcrowding of the plants.

### 2.3 Relative growth rate, Specific leaf area, and leaf dry matter content

Following Pérez‐Harguindeguy et al. (2016) protocol, we selected three relatively young (presumably more photosynthetically active) but fully expanded and hardened leaves from the upper and middle sections of the shoots from each aquarium (96 × 3 = 288 leaves). We measured the area of the leaves using a LI‐3000 Leaf area meter + LI‐3050C Transparent Belt Conveyor Accessory (LI‐COR Biosciences GmbH, Germany). The whole submerged plants and the three cut leaves were used for fresh weight and dry weight determination. We dried samples at 80°C for 48 h. After that, we immediately measured their weights on Ohaus Adventure Pro scale. The RGR of the plants was calculated as RGR = (ln*W*_t_ − ln*W*_0_)/*t*, where *W*_0_ represents the initial and *W*_t_ the final dry weight of the three plants in each aquarium, and t is the cultivation time in days.

We applied two additional traits in the subsequent analyses; both were calculated from the measured leaf area, leaf fresh weight, and leaf dry weight data.

Specific leaf area (SLA) was calculated as SLA = (LA/*W*_t_ mm^2^ mg^−1^) where LA represents the leaf area and W_t_ the dry weight of the leaves. We applied SLA because it tends to scale positively with mass‐based light‐saturated photosynthetic rate, and in general, species tend to have higher SLA in permanently or temporarily resource‐rich environments than do those in resource‐poor environments.

Leaf dry matter content (LDMC) was calculated as LDMC = (*W*_t_/*W*_0_ mg g^−1^), where W_0_ represents the initial (water saturated) and *W*_t_ the final dry weight of the leaves. Leaves with high LDMC tend to be relatively tough and are thus assumed to be more resistant to physical hazards.

### 2.4 Chemical composition

At the end of the experiment, we analyzed nitrogen and carbon concentration of the dried plants (96 samples) by dry combustion using a Vario Max Cube elemental analyzer (Elementar GmbH, Germany).

### 2.5 Plasticity index

We calculated the plasticity index (PI) for RGR, SLA, and LDMC for light (PI_L_), nutrients (PI_N_), and temperature (PI_T_) according to Szabó et al. (2019) as: PI = (maximum mean–minimum mean)/maximum mean. The index ranges from 0 (no plasticity) to 1 (maximum plasticity).

### 2.6 Statistical analysis

Normality of the variables was checked by the Kolmogorov–Smirnov test. RGR, SLA, and LDMC were all normally distributed (*p* > .05). A general linear model (GLM) was used to test the significance of the factors (light, nutrient, temperature, and species identity) and their interactions on the variables. Pairwise comparisons (PC) were used to test the variables for significant differences between species where mean difference (MD) ± standard error was indicated; furthermore, ANOVA was used to test the significance level when examining the effect of an independent variable on a dependent variable for a given species. All analyses were made using SPSS 16.0 software.

## 3 RESULTS

### 3.1 Relative growth rate

**FIGURE 1:**
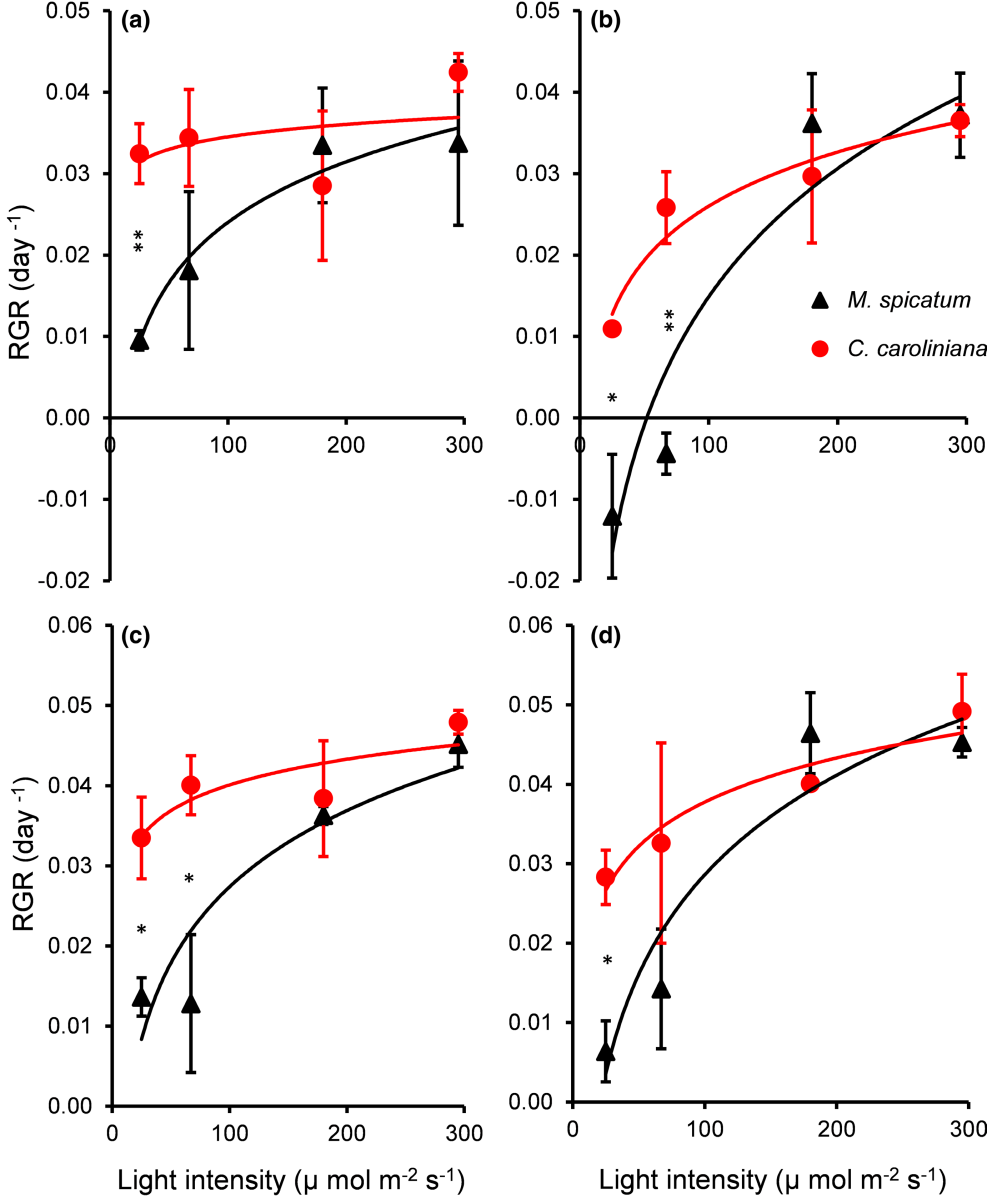
Relative growth rate (RGR [dry weight]) of *Myriophyllum spicatum* and *Cabomba caroliniana* cultures grown at different light levels and treatments (a) low nutrient and low temperature; (b) low nutrient, high temperature; (c) high nutrient, low temperature; (d) high nutrient and high temperature) (mean ± SE, *N* = 3). Asterisks indicate a significant difference (PC) between the species (*p < .05, **p < .01).

For the overall experiment, all independent variables (light intensity, nutrient concentration, and temperature) have a significant effect on the RGR of both species (Table S1). Increasing temperature significantly (MD = −0.011 ± 0.005, *p* = .018) reduced the RGR of submerged plants at low (L1 & L2) light intensity (Figure 1b, d). RGR of *M. spicatum* measured at high nutrient concentration was significantly higher (MD = 0.009 ± 0.003, *p* = .007) than at low concentration; nevertheless, it was not affected by temperature treatments. Nutrient concentration significantly influenced (*F* = 9.049, *p* = .005) the growth of *C. caroliniana*. For the overall experiment, *C. caroliniana* showed significantly higher RGR than *M. spicatum* (MD = 0.011 ± 0.003, *p* = .001) (Figures 1 and 5). Moreover, at lower light intensity (L1 and L2), the differences were even larger (MD = 0.022 ± 0.004, *p* < .001). However, at high light intensity (L3 and L4), there were no significant differences detected between growth rates of plant species (Figure 1).

### 3.2 Specific leaf area

**FIGURE 2:**
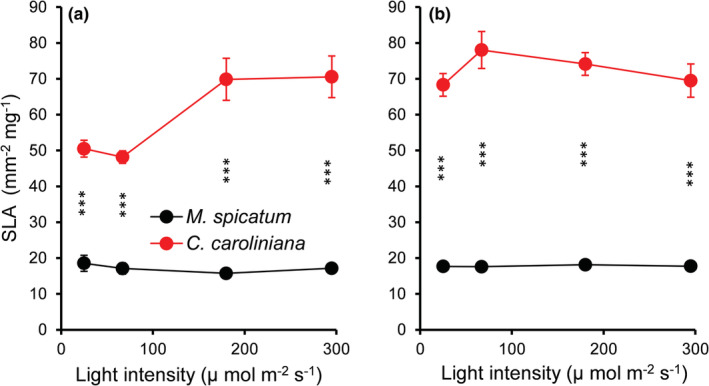
Specific leaf area (SLA mm^−2^ mg^−1^) of *Myriophyllum spicatum* and *Cabomba caroliniana* cultures grown at different light levels combined with low (a) and high (b) temperature. Each point represents data from low and high nutrient concentrations (mean ± SE, *N* = 6). Asterisks indicate a significant difference (PC) between the species (***p < .001).

For the overall experiment, light intensity, species identity, and temperature had a significant effect on the SLA, but nutrient concentration did not (Table S1). On one hand, SLA of *M. spicatum* was not altered at all neither by nutrient concentration, light intensity, or temperature. By contrast, SLA of *C. caroliniana* was affected both by light intensity (*F* = 3.413, *p* = .029) and temperature (*F* = 18.464, *p* < .001) (Figure 2). SLA of *C. caroliniana* was significantly higher at high temperature (MD = 13.1 ± 3.0, *p* < .001). Light intensity had a significant (MD = 20.0 ± 4.1, *p* < .001) effect on SLA of *C. caroliniana* only at low temperatures. In all cases, SLA of *C. caroliniana* was significantly (MD = 48.5 ± 2.0, *p* < .001) higher than that of *M. spicatum* (Figures 2 and 5).

### 3.3 Leaf dry matter content

**FIGURE 3:**
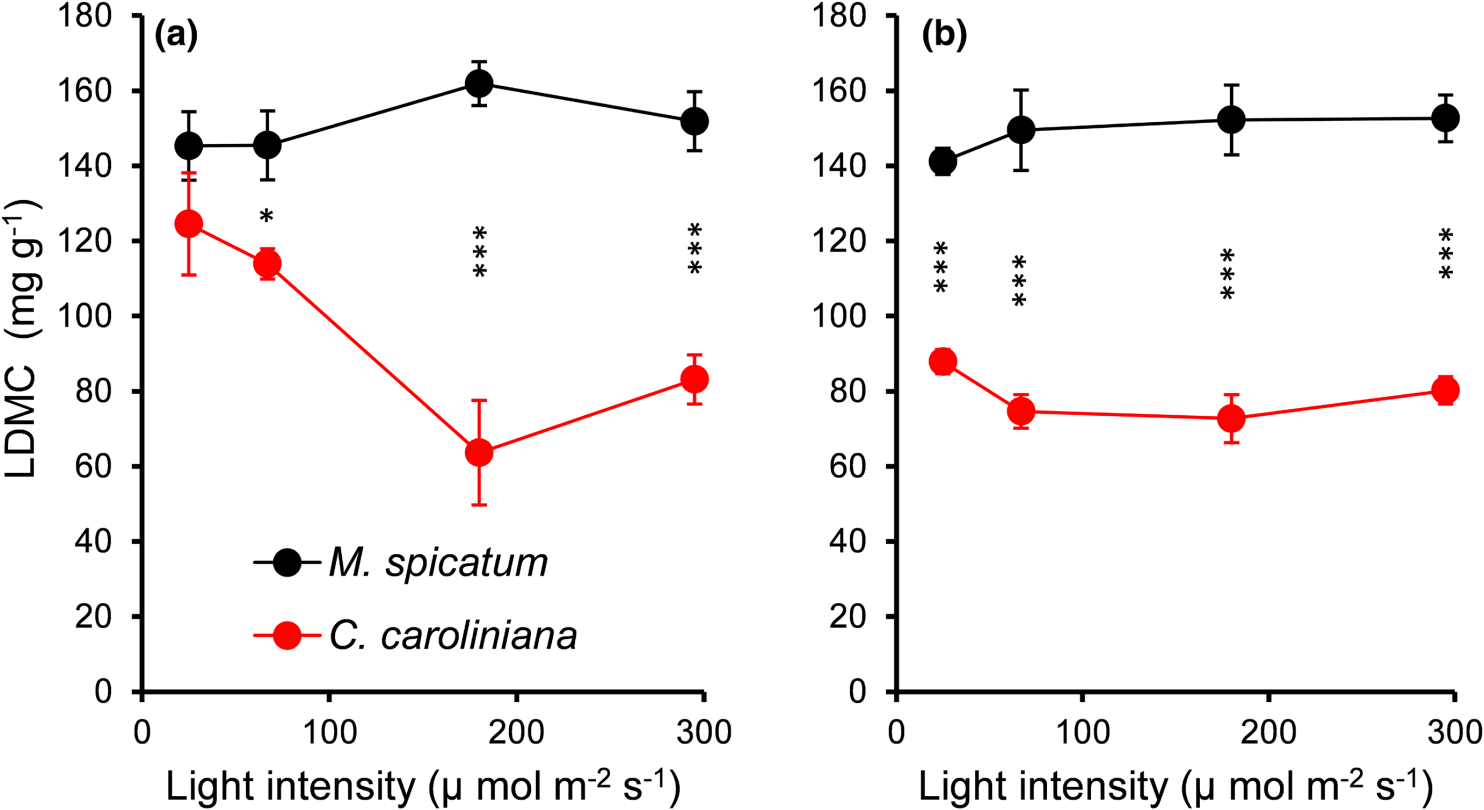
Leaf dry matter content (LDMC mg g^−1^) of *Myriophyllum spicatum* and *Cabomba caroliniana* cultures grown at different light levels combined with low (a) and high (b) temperature. Each point represents data from low and high nutrient concentrations (mean ± SE, *N* = 6). Asterisks indicate a significant difference (PC) between the species (*p < .05, ***p < .001).

Species identity and temperature had a significant effect on LDMC (Table S1). LDMC of *M. spicatum* was significantly higher (MD = 0.061 ± 0.004, *p* < .001) than that of *C. caroliniana* (Figures 3 and 5) in all light intensity treatments. However, the differences were not significant under low temperature (21.5 ± 0.5°C) combined with low light intensity (L1) (Figure 3). *Cabomba caroliniana* showed reduced LDMC value with higher temperature (MD = 0.020 ± 0.006, *p* = .002).

### 3.4 N:C molar ratio

**FIGURE 4:**
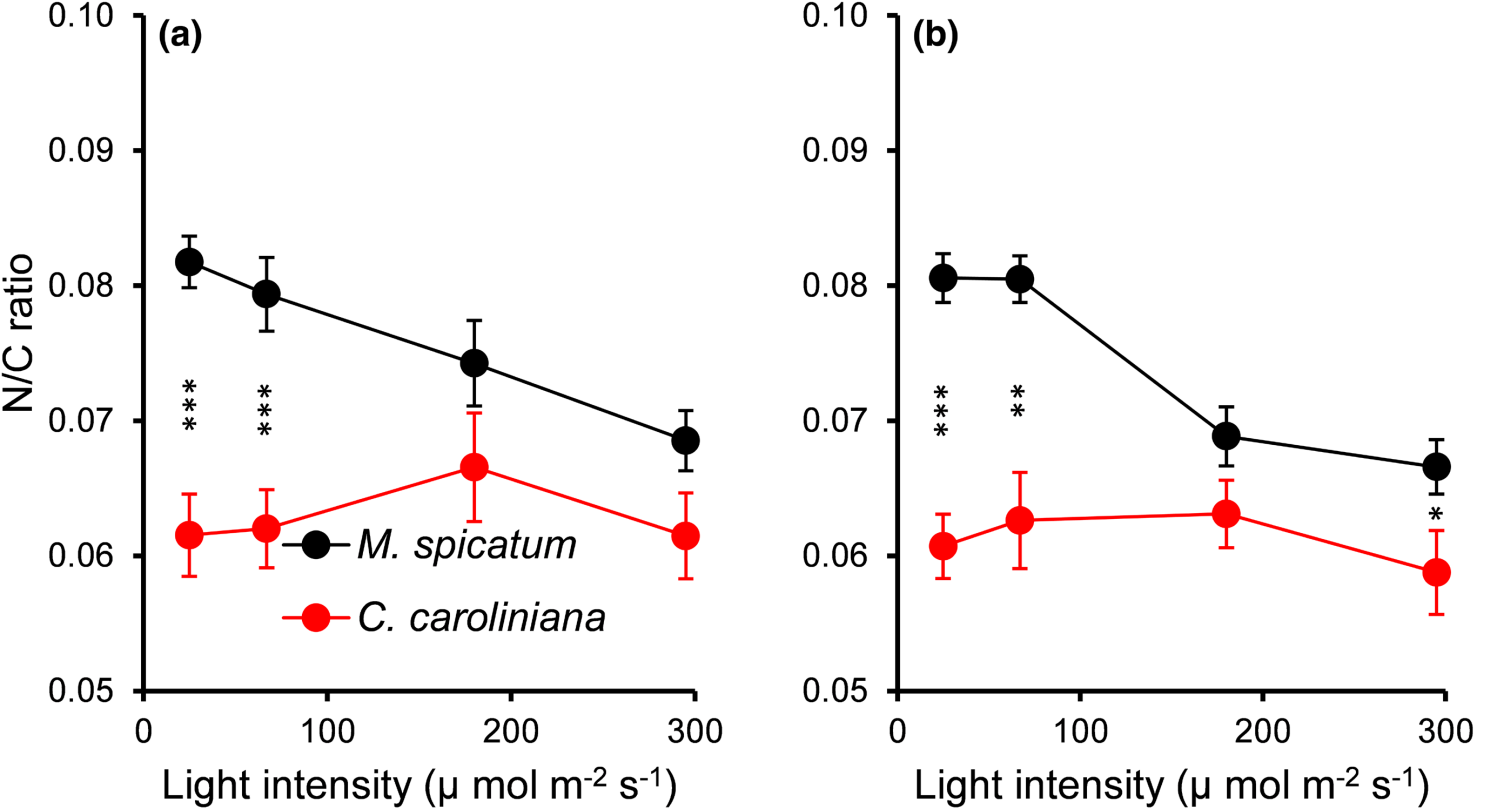
N:C molar ratio of *Myriophyllum spicatum* and *Cabomba caroliniana* cultures grown at different light levels combined with low (a) and high (b) temperature. Each point represents data from low and high nutrient concentrations (mean ± SE, *N* = 6). Asterisks indicate a significant difference (PC) between the species (*p < .05, **p < .01, ***p < .001).

**FIGURE 5:**
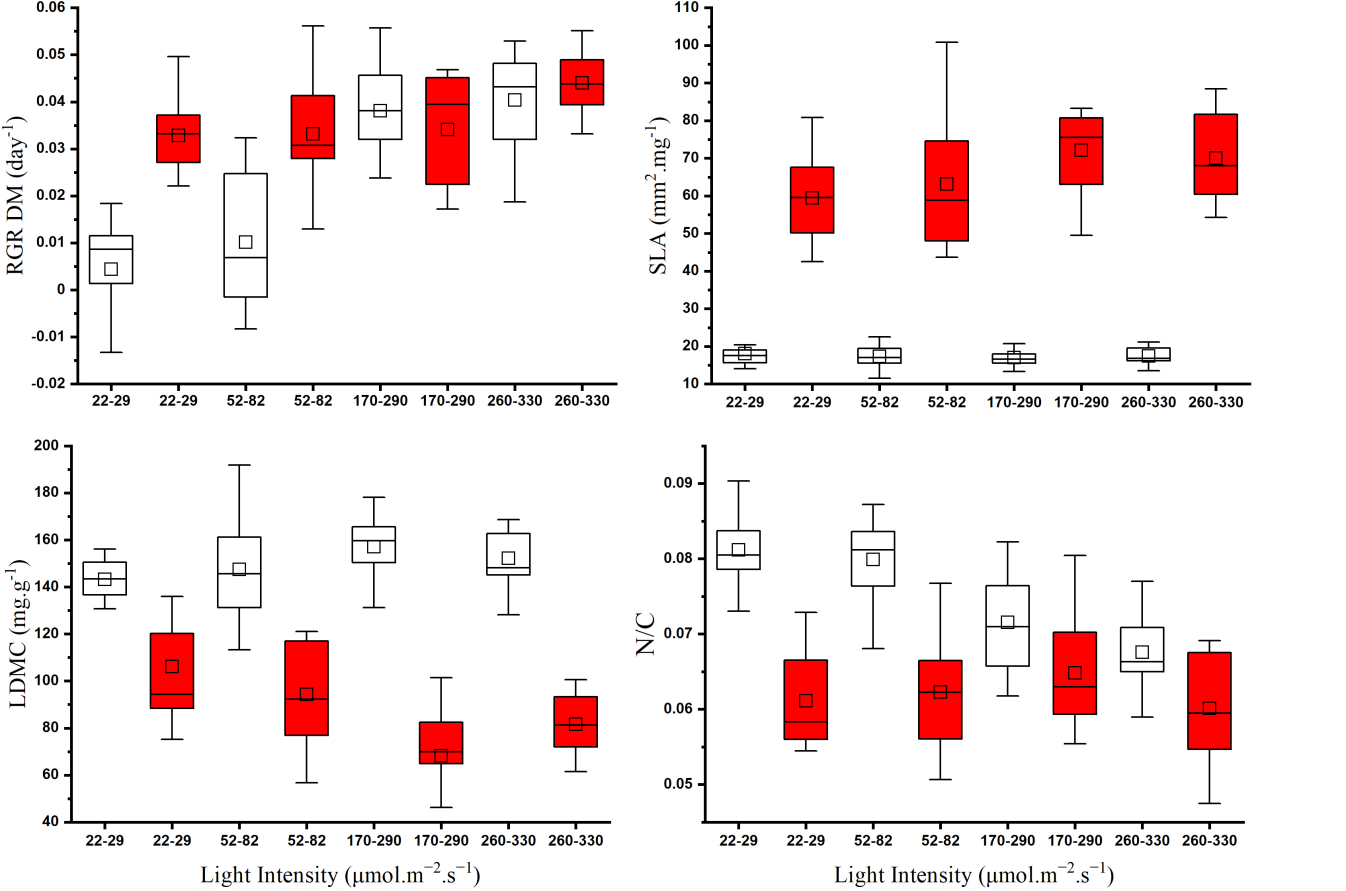
Boxplots of relative growth rate (RGR [dry weight]), specific leaf area (SLA), leaf dry matter content (LDMC), and N:C molar ratio of *Myriophyllum spicatum* (white) and *Cabomba caroliniana* (red) under different light levels. Each boxplot represents data of four light intensities. Boxes: +25%–75% percentiles; whiskers: Standard deviations, □: Median, *n* = 12.

**FIGURE 6:**
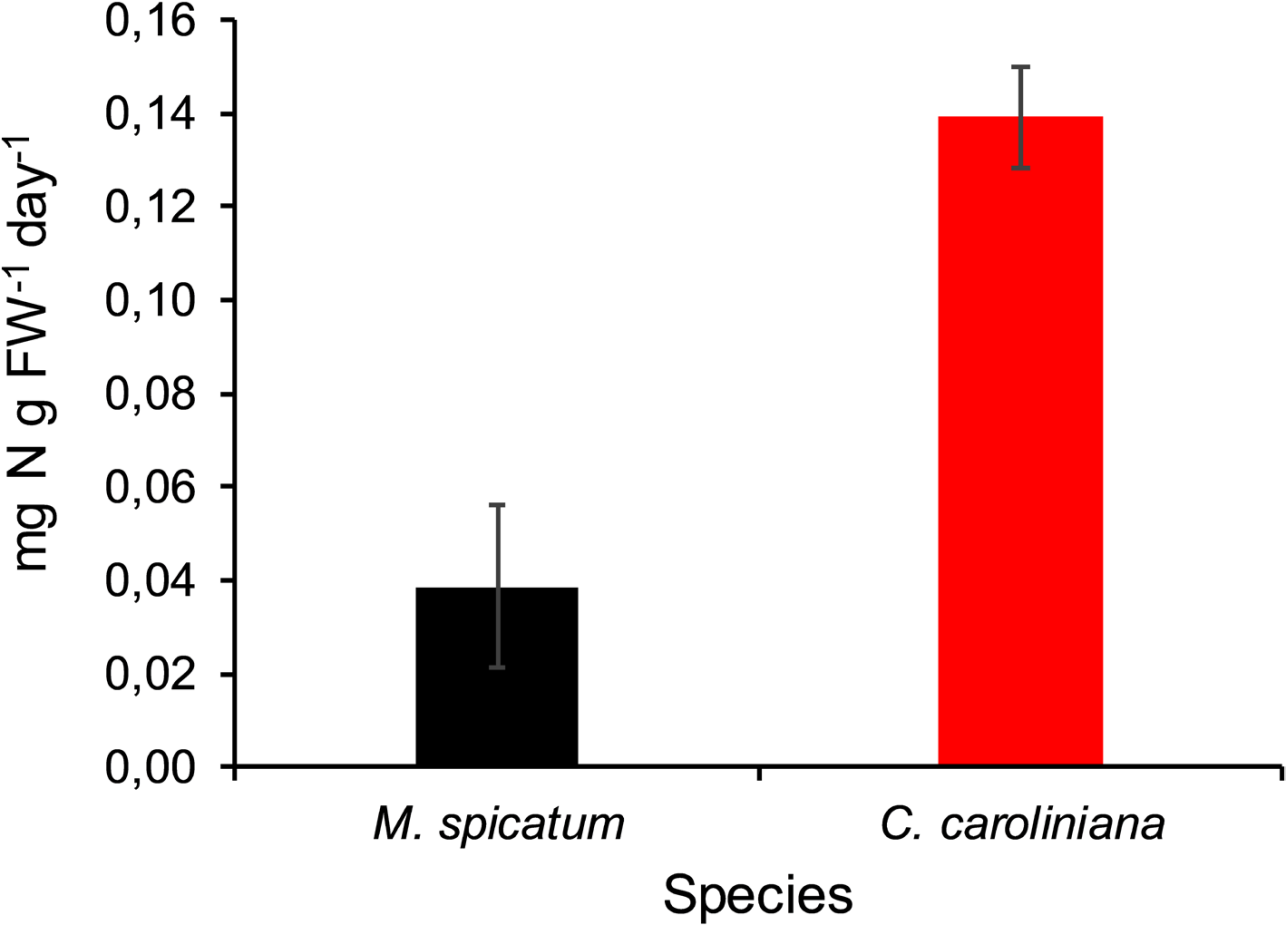
Nitrogen uptake (mg N g FW^−1^ day^−1^) of *Myriophyllum spicatum* and *Cabomba caroliniana* cultures grown at low light levels (L1 & L2) with high temperature. Each column represents data from low and high nutrient concentrations (mean ± SE, *N* = 12).

For the overall experiment, nutrient, light, and species identity had a significant effect on the N:C molar ratio (Table S1). Overall, *M. spicatum* had a significantly higher (MD = 0.013 ± 0.001, *p* < .001) N:C molar ratio than *C. caroliniana*; furthermore, under low light conditions (L1 and L2), the differences between species were even greater (Figures 4 and 5). On the other hand, at low light conditions (L1 and L2) with high temperature, the daily nitrogen uptake (mg N g FW^−1^ day^−1^) of *C. caroliniana* was significantly higher (MD = 0.100 ± 0.021, *p* < .001) and 3.6 times faster than that of *M. spicatum* (Figure 6).

### 3.5 Differences in phenotypic plasticity

Along the examined light gradient combined with temperature and nutrient levels, the two submerged species showed marked differences in their phenotypic plasticity (Table 1). On one hand, *M. spicatum* showed greater (by 0.1) plasticity for light regarding to RGR. On the other hand, *C. caroliniana* showed higher (by 0.1) plasticity for light and temperature in SLA and LDMC. With regard to nutrients, the two species did not show any characteristic differences in their phenotypic plasticity values. Regarding the overall phenotypic characteristics, *C. caroliniana* showed a higher plasticity than *M. spicatum*.

**TABLE 1:** Plasticity index (PI) of *Myriophyllum spicatum* (*Myrioph.*) and *Cabomba caroliniana* (*Cabomba*) for light (PI_L_), nutrients (PI_N_), and temperature (PI_N_). Greater PI values are bold if differed by 0.1.

Variable	*Myrioph*.	*Cabomba*	*Myrioph*.	*Cabomba*	*Myrioph*.	*Cabomba*
	PI _Light_		PI _Nutrient_		PI _Temperature_	
RGR	**0.89**	0.40	0.31	0.22	0.17	0.15
SLA	0.07	**0.20**	0.07	0.01	0.04	**0.18**
LDMC	0.06	**0.29**	0.05	0.05	0.01	**0.21**

## 4 DISCUSSION

The search for invasive traits and the investigation of trait plasticity constitutes a challenging task in freshwater biology and invasion biology. The best progress toward a general conclusion of this issue would be to pool evidence from pairwise comparisons and multispecies studies (Pysek & Richardson, 2007). In the case of aquatic plants, comparisons within growth forms have high relevance due to the scarcity of congeneric alien‐native species pairs. Since growth forms are stem from certain combination of traits, it can be conceptualized as groups of plants with similar degree of adaptation (Rowe & Speck, 2005). Anchored submerged plants with long stems and finely divided submerged leaves (i.e., Myriophyllid growth form) are successful in colonizing flowing waters (Wiegleb, 1991), since finely divided leaves give extra competitive advantages over species with entire leaves (Givnish, 1987). These advantages is due to the fact, that divided leaves have much more light capturing surface per unit biomass than entire leaves, and thereby they can be more efficient in photosynthesis. In consideration of this and its high natural dispersal potential, it is not surprising that *Cabomba caroliniana* is among the most successful invasive aquatic plants across Europe (Hussner, 2012).

Our laboratory results supported the idea that competitive success of the alien *C. caroliniana* comes from its higher specific leaf area (SLA) and 2–3 times higher growth rate under shaded conditions than *M. spicatum*, which indicated that *C. caroliniana* tolerated the shade better (more shade tolerant). Under shaded conditions, higher temperature induced even higher specific leaf area (SLA) for *Cabomba*. Therefore, our results are in line with the finding of Lukács et al. (2017) who pointed out that among *Myriophyllids* the invader species had higher SLA than natives. Furthermore, it also supports the findings of Lake and Leishman (2004) and Hamilton et al. (2005) who found that high SLA can promote invasiveness.

In addition, higher SLA and lower LDMC of *Cabomba* versus *Myriophyllum* suggest that *Cabomba* can form much more leaf area from the same dry matter content (LDMC), which can also contribute to its invasion success. Invaders having lower LDMC may have an advantage in the competition for light, because softer leaf tissues allow invaders to build their photosynthetic organs faster and invest less into structural tissue elements (Lukács et al., 2017). Our results support those findings revealing that some invader species can invest much more on relative shoot elongation (mm mg^−1^ DW) which can provide a better position for light capture (Szabó et al., 2019). It also suits the finding that alien species can develop larger leaf area faster, thereby increasing their invasion success (Lukács et al., 2017).

Since former studies have already pointed out that *Cabomba* has high light requirements (Hiscock, 2003; Scheurmann, 1993), it was expected that low light intensity would have a limiting impact on its growth. However, in our experiment, *Cabomba* showed much less reduction in growth at low light levels than did *Myriophyllum*. Additionally, the growth rate of *Cabomba* was two–three times higher than that of *Myriophyllum*, indicating its lower light compensation point compared with *Myriophyllum*. Under natural conditions, especially in eutrophic waters, epiphytic algae can shade submerged plants by up to 90%, decreasing their photosynthesis and growth (Bulthuis & Woelkerling, 1983; Koleszár et al., 2021; Phillips et al., 2016; Tóth, 2013). However, both species are able to produce allelopathic substances against blue green algae, thereby partly lowering the shading effect of periphyton (Nakai et al., 1999). In this way, those species equipped with a trait such as lower light compensation point might gain extra competitive advantage. Our results indicate that *Cabomba* can be more competitive species than *Myriophyllum*, and it may survive better under more shaded eutrophic conditions, such as turbid water or below a mat of floating plants (Szabó et al., 2020; van Gerven et al., 2015).

In our study, higher nutrient concentration only slightly enhanced the RGR of *Myriophyllum*, and *Cabomba*. On the contrary, analyzing the tissue N concentration revealed that under low light intensity, *Cabomba* has more than three times higher nitrogen uptake capacity than *Myriophyllum* (Figure 6). This trait may be especially advantageous in waters with suboptimal nitrogen concentration. Furthermore, at low light intensity, increasing temperature reduced the growth rate of both submerged plants, but significantly enhanced the SLA of *Cabomba* and reduced its LDMC likely contributing to its invasion success.

The aim of our study was to investigate the combined effects of temperature, light, and nutrients (N, P) on the trait responses (RGR, SLA, LDMC, N:C molar ratio) of the two species in an 8‐day laboratory experiment. However, several questions may arise regarding the duration of the experiment. On the one hand, the plants were properly preincubated since shoot length and biomass nearly doubled in the 14 days of preincubation (via RGR 0.04 day^−1^); thus, after cutting the apical end, newly developed shoots were used for the experiment. On the other hand, this period was sufficient to find well‐detectable significant changes in the plant traits due to the various light intensities and/or nutrient concentrations and/or temperature levels, as mentioned above. Although an 8‐day period experiment may seem relatively short, the reason we finished the experiment after 8 days was to avoid overcrowding of the plants in 2‐L aquaria. In case of overcrowding, the changes in traits are no longer just due to the treatments (light, nutrient, and temperature), but also due to self‐shading (intraspecific competition). Our results under noncrowded laboratory conditions with low light levels partly imitated those field conditions with densely growing shaded plants where the invasion is actually happening. Thus, the measured growth traits along light gradient gave relevant information for field conditions as well. Over and above, there are several studies (Cedergreen et al., 2004; Huang et al., 2017; Szabó et al., 2019, 2020) clearly supported that 14 days of preincubation with 8‐day incubation was sufficient to compare traits (RGR, LDMC, N:C molar ratio, SLA) between aquatic plant species.

Our short‐term experimental results pointed out that the three environmental factors significantly modified the investigated plant traits. However, it is well known, that these abiotic conditions constantly change over longer growing periods. Due to apical elongation of the shoots, it is obvious that light intensity increases with decreasing water depth (Pokorný et al., 1984). On the contrary, under hypertrophic conditions, shading of epiphytic algae can strongly decrease light conditions on the surface of the older leaves (Levi et al., 2015; Phillips et al., 2016; Tóth, 2013). Bioturbation of benthic fauna may not only decrease light conditions, but also increase nutrient release from the sediment to the water body as well (Adámek & Maršálek, 2013; Chen et al., 2016; Scheffer, 1998). By contrast, in stands of submerged vegetation due to plant nutrient uptake, nutrient concentration (N, P) of the water shows continuous decrease over the growing season (Scheffer, 1998; Szabó et al., 2022). Beyond the increasing temperature over the growing season, submerged vegetation itself can strongly modify the water temperature due to reduced turbulence and shading. Thus, over a longer growing period, the differences in temperature between the upper and lower water bodies are increasing. All in all, even in the absence of crowding of the vegetation, both abiotic (light, nutrient, and temperature) and biotic conditions are constantly changing, together with the inevitable change of physiological condition of the aging plants. Thus, in field conditions, it is not at all likely that the determined traits will remain stable over a longer growing periods. By contrast, in our experimental setup, under a shorter incubation period, we were able to keep the environmental factors nearly constant in order to gain relevant information regarding to the plant traits under changing environment in field conditions. Obviously, our experimental results may differ in several ways from the results of long‐term studies under natural conditions. Natural pests and consumers may significantly change the competition between the two species (Koleszár et al., 2021). Furthermore, canopy formation of submerged macrophytes takes place along an increasing light gradient (from the shady bottom to the water surface); therefore, horizontal spread of the two species strongly depends on their apical elongation and branching degree along increasing light gradient (Szabó et al., 2019). Consequently, the differences in their phenotypic characteristics under various environmental conditions may also strongly determine their competitive outcome. Therefore, it is obvious, that our laboratory results do not directly reflect the complexity of field conditions therefore, may not directly indicate the invasion success of *Cabomba*. However, the change in the observed traits in these controlled conditions is well consistent with the already‐documented invasion of *Cabomba*. Thus, this study may help to understand further study of field traits of these species under more complex conditions.

Comparing all the results we may conclude, that H1 hypothesis was supported by the results that the trait differences for RGR and N:C molar ratio between species were more pronounced under decreasing light conditions and this likely contribute to the invasion success of *Cabomba* over *Myriophyllum*. Since *Myriophyllum* showed higher phenotypic plasticity for RGR, and *Cabomba* for SLA and LDMC, our second hypothesis has been proved to be also partly true. However, these characteristics make *Cabomba* a better survivor under turbid eutrophic conditions, contributing to the rapid spread of the species.

Based on these findings, shade tolerance seems to be a key factor in the invasion success of *Cabomba caroliniana*. In order to reveal more realistic image of the interplay between the two species, further long‐term mesocosm experiments are needed to be performed cultivating them in cocultures.

## AUTHOR CONTRIBUTIONS

**Gergő Koleszár:** Data curation (lead); formal analysis (lead); investigation (lead); methodology (equal); project administration (equal); software (equal); supervision (lead); validation (equal); visualization (lead); writing – original draft (lead); writing – review and editing (lead). **Balázs András Lukács:** Conceptualization (lead); data curation (supporting); formal analysis (lead); funding acquisition (lead); investigation (equal); methodology (supporting); project administration (equal); resources (lead); software (equal); validation (lead); visualization (equal); writing – review and editing (supporting). **Péter Tamás Nagy:** Data curation (supporting); methodology (supporting); software (lead). **Sándor Szabó:** Data curation (equal); formal analysis (equal); investigation (equal); methodology (equal); project administration (equal); software (supporting); validation (supporting); writing – review and editing (supporting).

## Supporting information


Table S1

## Data Availability

The datasets related to this study are available at https://datadryad.org/stash/share/2U0gbuswNQMg0AIIkj70mv0jhwzfjZTOP7jbh8XHhPg.
