# Supplementary material for: Shade tolerance as a key trait in invasion success of submerged macrophyte Cabomba caroliniana over Myriophyllum spicatum
Source: Ecol Evol. 2022 Sep 16;12(9):e9306. doi: 10.1002/ece3.9306 (PMC9481886; doi:10.1002/ece3.9306)
Supplement: Supplementary file 1 — Table S1 [file ECE3-12-e9306-s001.docx]

**Table S1.** Analysis of variance of the relative growth rate (RGR), specific leaf area (SLA), leaf dry matter content (LDMC) and N:C molar ratio of *Cabomba caroliniana* and *Myriophyllum spicatum* grown in aquaria under different light, nutrient and temperature levels. The Source/Trait abbreviations are as follows: N (Nutrient), L (Light), S (Species), T (Temperature).

| Source/Trait | *df* | F | Sig. |
| --- | --- | --- | --- |
| **RGR** |  |  |  |
| N | 1 | 17.40 | **<0.001** |
| L | 3 | 36.90 | **<0.001** |
| S | 1 | 29.43 | **<0.001** |
| T | 1 | 5.50 | **0.022** |
| N * T | 1 | 4.33 | **0.041** |
| L * S | 3 | 10.67 | **<0.001** |
| L * T | 3 | 3.85 | **0.014** |
| **SLA** |  |  |  |
| L | 3 | 2.77 | **0.049** |
| S | 1 | 935.21 | **<0.001** |
| T | 1 | 18.66 | **<0.001** |
| L * S | 3 | 3.55 | **0.019** |
| L * T | 3 | 4.15 | **0.009** |
| S * T | 1 | 15.41 | **<0.001** |
| L * S * T | 3 | 4.99 | **0.004** |
| **LDMC** |  |  |  |
| S | 1 | 247.14 | **<0.001** |
| T | 1 | 9.09 | **0.004** |
| L * S | 3 | 6.32 | **0.001** |
| S * T | 1 | 5.11 | **0.027** |
| **N:C molar ratio** |  |  |  |
| N | 1 | 48.49 | **<0.001** |
| L | 3 | 10.38 | **<0.001** |
| S | 1 | 116.49 | **<0.001** |
| N * S | 1 | 4.36 | **0.041** |
| L * S | 3 | 6.02 | **0.001** |
